# A genetic algorithm enabled ensemble for unsupervised medical term extraction from clinical letters

**DOI:** 10.1186/s13755-015-0013-y

**Published:** 2015-12-09

**Authors:** Wei Liu, Bo Chuen Chung, Rui Wang, Jonathon Ng, Nigel Morlet

**Affiliations:** The University of Western Australia, 35 Stirling Highway, Crawley, WA 6009 Australia; Western Eye Clinic, Unit 5, 592 Stirling Highway, Mosman Park, WA 6012 Australia

**Keywords:** Clinical term extraction, Sequence mining algorithms, Genetic algorithm

## Abstract

Despite the rapid global movement towards electronic health records, clinical letters written in unstructured natural languages are still the preferred form of inter-practitioner communication about patients. These letters, when archived over a long period of time, provide invaluable longitudinal clinical details on individual and populations of patients. In this paper we present three unsupervised approaches, sequential pattern mining (PrefixSpan); frequency linguistic based C-*Value*; and keyphrase extraction from co-occurrence graphs (TextRank), to automatically extract single and multi-word medical terms without domain-specific knowledge. Because each of the three approaches focuses on different aspects of the language feature space, we propose a genetic algorithm to learn the best parameters of linearly integrating the three extractors for optimal performance against domain expert annotations. Around 30,000 clinical letters sent over the past decade from ophthalmology specialists to general practitioners at an eye clinic are anonymised as the corpus to evaluate the effectiveness of the ensemble against individual extractors. With minimal annotation, the ensemble achieves an average F-measure of 65.65 % when considering only complex medical terms, and a F-measure of 72.47 % if we take single word terms (i.e. unigrams) into consideration, markedly better than the three term extraction techniques when used alone.

## Background

The amount of electronic descriptive clinical documents generated by medical practitioners at various levels of expertise is enormous, easily reaching zettabytes [1]. It is expected to grow even more as computing devices’ processing power and storage become increasingly accommodating. These clinical documents may include texts such as patient records, clinical notes, discharge summaries, doctors’ referral letters and so forth. Accompanying this exponential growth of electronic medical documents is the very urgent need of techniques to process them into meaningful information that can support the advancement of medical science and practice.

Unsurprisingly, clinical documentation today is still mostly written in unstructured natural language formats as opposed to structured database records. Looking into the foreseeable future, unstructured natural language text will likely remain the preferred form of clinical communication due to its flexibility and much reduced disruptions to doctors’ daily routines. In fact, a study carried out by IBM [2] in 2013 predicted that nearly 80 % of medical data will be in unstructured textual format by 2015. These documents contribute a rich resource to support research such as epidemiological studies, treatment effectiveness analysis, and medical decision making, just to name a few. Natural Language Processing (NLP), with its recent success in information and entity extraction, provides a promising solution space for annotating and structuring text-based clinical information into databases thus making them readily retrievable and analysable for health professionals. In doing so, the costs of producing structured medical records can be reduced, along with improved overall quality and accuracy [3].

Most of the work on biomedical natural language processing [4–6], focus on *medical entity extraction* and *assertion classification*. Take the sentence No active bleeding was observed for example, active bleeding would be extracted as a medical entity, and Absent would be the class label for the entity assertion. The problem we are interested in is *medical entity extraction* or more precisely *medical term extraction*, which identifies the medical terms or concepts occurring in clinical documents *without* the identification of meta-level entity types.

The medical term extraction task alone already is a challenging one, because most of the state-of-the-art clinical term recognition systems are based on supervised machine learning techniques, requiring a significant amount of manual effort for producing the training dataset. In general, a supervised technique involve three tasks, feature collection, training dataset labelling, and classifier model development. The most common features used are dictionary lookup, bag of words, Part Of Speech (POS) tags, window size, orientation, distance and capitalisation. After feature collection, the non-trivial and often the bottleneck task is to manually label the datasets into pre-defined categories or classes, which is not only time-consuming but also error-prone. As an example of supervised approaches, Wang et al. [7] combined rule-based techniques with Conditional Random Fields (CRF) to annotate clinical notes containing informal clinical terminologies.

To overcome the difficulties in manual labelling such large amounts of document sets, in this paper, we investigate *unsupervised approaches in medical term extraction*. The most basic unsupervised approach is dictionary look-up. For example, the MetaMap Transfer (MMTx) software tool^1^, offered by National Institutes of Health (U.S.), makes use of a medical term thesaurus ULMS^2^ for recognising medical concepts in text. Advanced unsupervised approaches would mostly rely upon pre-defined rules for noun phrase chunking [8] or different ways of combining statistical and linguistic cues (i.e. C-*Value* and its extension NC-*Value*) [9–11].

Applying sequential data mining techniques on medical entity extraction has only recently attracted the attention of the NLP community thanks to the very recent work of Liu et al. [12] and Ren et al. [13]. Our work is conducted in parallel with this thread of work on applying frequent sequence mining algorithms for phrase extraction. In this paper, the PrefixSpan algorithm [14] is adapted as the first unsupervised approach for medical concept extraction. The second unsupervised approach is C-*Value* [9], where both statistical and linguistic information are taken into account. In the third approach, we consider the medical term extraction as a keyphrase extraction or document summarisation task, through analysing co-occurrence graph using the popular TextRank algorithm [15].

We hypothesise that the three techniques are complementary, as each focuses on different aspects of the language feature space. Sequence mining relies on the fact that words in complex medical terms often occur in order, which is at the *lexical level*. C-*Value* requires *syntactical level* part of speech filtering to identify noun phrases and their frequency and sub-term frequency, whereas TextRank pays more attention to graph based *structural level* co-occurrence relations. To make best use of all three ranking scores, we developed a Genetic Algorithm (GA) to learn the weights for a linear combination of the scores generated from each of the three algorithms. Our approach has general applicability to other term extraction algorithms so long as they are able to produce ranking scores. For evaluation we collected and processed 29,232 clinical letters from Western Eye Specialists Clinic^3^. All three algorithms and the GA-enabled ensemble were tested and evaluated on this corpus. With minimal annotation, the ensemble achieve an average F-measure of 65.65 % when considering only complex medical terms, and a F-measure of 72.47 % if we take single word terms (i.e. unigrams) into consideration. This represents marked improvement on the performance of individual algorithm alone.

The paper is organised as follows. The “Related work” section provides an overview on the related work in unsupervised automatic medical term extraction. The “Methodology” section explains the three different medical concept extraction techniques used in this research, and how an ophthalmology dictionary can be built from online resources for verification and filtering purposes. After that, the “Genetic algorithm enabled ensemble” section describes the process of designing a genetic algorithm to combine the ranking scores from the three term extraction algorithms. The "Experiments" section provides information about the parameter settings of different algorithms while results are reported in the “Results and discussion” section. The paper concludes with an outlook to future work in the “Conclusion” section.

## Related work

Automated Term Extraction is the process of using computer software to automatically identify and extract strings that are potential domain-specific terms from a collection of documents [16]. Many different approaches have been developed for automated term extraction, including linguistic approaches [17, 18], statistical approaches [19, 20], or a combination of both [21]. Two major steps are involved, namely candidate term generation and statistical analysis to rank the candidates.

### Candidate term generation

Two main approaches often adopted for candidate term generation include:

- *Linguistic Filters* This requires tagging the words in a document with part of speeches (e.g. nouns, verbs, adjectives, etc.). The terms are then extracted using a regular expression pattern filter, which specifies the sequences of part of speech that are considered possible terms. For example, the filter might specify that only noun phrases (sequence of nouns) are considered possible candidate terms and thus all other non-noun words are filtered out.
- *N-gram* An *n*-gram is defined as a continuous sequence of *n* words from a text. The candidate terms are then extracted by generating all *n*-grams from the text for *n* = 1,2,..., *k* , where *k* denotes the maximum length of a term. For example, if the text is “Dog chase cat” and the maximum length of a term is two, then the *n*-gram approach will generate the terms “*dog chase*”, “*chase cat*”, “*dog*”, “*chase*” and “*cat*” as possible candidate terms.

### Statistical analysis

After the list of candidate terms are generated, statistical analysis is performed to rank the candidate terms according to certain measures such that higher scoring terms are more likely to be actual terms than lower scoring ones. An empirical threshold is commonly used to specify the cut-off point. Frequency is one of the most widely used measures due to its reliability in identifying terms [22]. The basic assumption is that if a candidate term appears frequently enough in a corpus, then it is more likely to be an actual valid term. For example, if the candidate term “*visual acuity*” appears in a corpus 50 times and the minimum frequency threshold is 10, then “*visual acuity*” will be selected and returned by the term extractor as final terms.

However, frequency alone is often not enough. Therefore, more advanced measures such as *t-score*, *log-likelihood*, *mutual information* and $\chi ^{2}$ (Chi-squared), which we describe below.

Before that, we need to describe a contingency table, shown in Table 1, which will be used in all the measures mentioned above. The table shows that observed frequencies of any word pair (or collocation) between individual words *a* and *b*. $\overline{a}$ represents any word that is not *a* and $\overline{b}$ represents any word that is not *b*. However, in order to extract terms, an ordering between *a* and *b* must be enforced. Thus *ab* represents a term where *b* is immediately preceded by *a* and not the occurrence of *a* and *b* together without any ordering. Thus in the examples below, *ab* is not equivalent to *ba*. The frequency for *ab* is represented by $n_{11}$ while $n_{pp}$ is the total number of word pairs in the corpus, calculated by $n_{pp}$ = $n_{1p}$ + $n_{2p}$ + $n_{p1}$ + $n_{p2}$. Marginal and expected frequencies can also be calculated from the table. For example, the marginal frequency $n_{1p}$ is the frequency of all word pairs that start with *a* while the expected frequency $m_{11}$ of *ab* is given by $\frac{n_{p1} \cdot n_{1p}}{n_{pp}}$ [16].

**Table 1:** Contingency table for word pair *ab*

	*a*	$\overline{a}$	
*b*	$n_{11}$	$n_{21}$	$n_{p1}$
$\overline{b}$	$n_{12}$	$n_{22}$	$n_{p2}$
	$n_{1p}$	$n_{2p}$	$n_{pp}$

#### T-score

*t-score* does not measure the statistical strength of association between *a* and *b* in the word pair *ab*, but it provides the confidence for which we can assert whether *a* and *b* can actually co-occur together as a term. The formula for calculating *t-score* is given below [16]:

$$\begin{aligned} \textit{t-score}(a,b) = \frac{n_{11} - m_{11}}{\sqrt{n_{11}}} \end{aligned}$$

The null hypothesis that *a* and *b* does not have any significant association and are independent of one another is commonly used in the *t-score* technique. Thus, if the *t-score* is greater than the critical value $\alpha$ for a given confidence interval, we can reject the null hypothesis and conclude that there exists an association between *a* and *b* and that both words together reasonably form a valid term.

#### Mutual information

 Mutual information (MI) measures the mutual dependence or the information shared between the two words *a* and *b*. The formula for calculating mutual information is given below [23]:

$$\begin{aligned} MI(a,b) = \log _{2}\frac{p(a,b)}{p(a) p(b)} \end{aligned}$$

In the context of the contingency table, *p*(*x*, *y*) is equivalent to $n_{11}$, the observed frequency of the word pair *ab* while *p*(*a*) and *p*(*b*) refers to the marginal frequency of *a* and *b* respectively, $n_{1p}$ and $n_{p1}$. Intuitively, mutual information compares the frequency of the word pair *ab* against the frequency of the individual component words *a* and *b*. Thus if a word pair has a high frequency compared to its component words, then by mutual information, it is very likely that *ab* is a valid term.

#### Log-likelihood

 The log-likelihood (LL) approach uses a ratio test to determine the statistical significance of association between *a* and *b*. The approach computes the likelihood of the observed frequencies under two hypotheses, the null hypothesis $H_{0}$ that states *a* and *b* are independent and the alternative hypothesis $H_{1}$ that states there is an association between *a* and *b* [24]. The two hypotheses’ likelihoods are then compared and combined into a single ratio, which gives a larger value if there is a strong association between *a* and *b*. The formula for the log-likelihood ratio is given below [16]:

$$\begin{aligned} LL(a,b) = 2 \sum \limits _{ij}^{} n_{ij} \log \frac{n_{ij}}{m_{ij}} \end{aligned}$$

where *i* ranges over the rows and *j* over the columns of the contingency table in Table 1.

$\chi ^{2}$*(Chi-squared)* The Chi-squared technique compares the observed frequency of *ab* against its expected frequency to test the null hypothesis that *a* and *b* are independent. If the observed frequency is much greater than the expected frequency, the null hypothesis of independence is then rejected [24]. The formula for $\chi ^{2}$ is given below:

$$\begin{aligned} \chi ^{2} (a, b) = \sum \limits _{ij}^{} \frac{(n_{ij} - m_{ij})^{2}}{m_{ij}} \end{aligned}$$

#### TextRank

TextRank was introduced by Mihalcea and Tarau [15], which is a graph-based ranking algorithm for keyphrase extraction and text summarisation. It first constructs an un-weighted undirected graph representing a given document and then uses an algorithm detailed in the “Methodology” to rank how likely a pair of words form a complex term. In our recent work, Wang et al. [25, 26] investigated on how the processing steps and the incorporation of word embedding vectors into the weighting schemes affect its performance on key phrase extraction.

However, there is a major limitation for all the above methods as they only work for word pairs (two words). In order to facilitate finding and extracting terms of more than two words, we need to first extract out all the valid two-word pairs, tag them as a single word and rerun the methods. Thus the new word pairs might then consist a compositional (multi-worded) component. This can be computationally intensive as several passes of the corpus need to be performed in order to extract terms of longer length.

Fahmi [16], in the context of automatic medical question answering system, evaluated and compared several medical term extraction techniques on a Dutch medical corpus, including T-Score, Log-likelihood, Chi-squared and C-*Value*. Among these, the Chi-squared is reported, on average, the best performing technique.

Specific to medical documents, noun phrase chunking is often the first step used in medical term extraction. For example, Conrado et al. [8] demonstrated that it is possible to extract valid medical terms from a Spanish health and medical corpus by applying manually designed linguistic filters. A linguistic filter is a part of speech pattern specific to the language of interest. Three different noun phrase linguistic filters are designed and used. However, these rules are language specific and only capable of extracting unigram, bigram and trigram medical terms. For evaluation, the extracted candidate terms are compared against IULA medical reference list. It is demonstrated that the linguistic filters are able to extract terms not present in the list. Manually validated terms are then used to expand the Spanish SNOMED CT^4^.

#### C-*Value*

C-*Value* and its extension, NC-value developed by Frantzi et al. [9], after noun phrase chunking, produces a unithood score based on the length of the phrase as well as the phrase and sub-phrase frequency to rank the candidate terms. NC-*Value* also incorporates contextual information surrounding the terms to improve the term extraction accuracy and quality of the term extracted. They are also able to arbitrarily extract terms of any length.

However there are no mathematical justifications on why the phrase and sub-phrase frequencies are combined in the proposed way. To address such issues, in our previous work, Wong et al. [10, 11] developed a probabilistic framework to combine evidence of how exclusive and prevalent a term occurs in a domain corpus in contrast to general corpora.

Having said that, C-*Value* and NC-*Value* are still the most widely used unsupervised phrase extraction techniques by far as it requires domain corpora only. For example, in the popular downstream task of large scale medical document indexing, by applying C-*Value* and NC-*Value* as a crucial part of their AMT$_{X}$ system, Hliaoutakis et al. [27] shows that AMT$_{X}$ outperforms MMTx in both precision and recall. As mentioned in the Introduction section, MMTx automatically maps biomedical documents to UMLS concepts through dictionary look-up.

#### Sequential pattern mining

Frequent sequence mining has only recently gain attentions in entity extraction due to (1) its speed in dealing with massive text corpora; (2) its language-neutral property as there is no need in formulating language-specific linguistic rules; (3) its minimal requirement on training data. Plantevit et al. [28] developed left-sequence-right (LSR) patterns to search for named entities from real datasets BioCreative, Genia and Abstracts, taking into account the surrounding context of a sequence and relaxing the order constraint around the sequence. The presence of sequential pattern mining algorithms in phrase extraction from massive text corpora by Liu et al. [12] and Ren et al. [13] in 2015 will certainly promote more research on medical term extraction to adopt such an approach.

## Methodology

The three techniques implemented for this paper are: PrefixSpan, a *n*-gram frequency based extractor; C-*Value*, a linguistic and statistical term extractor; and TextRank, a graph based co-occurrence analysis algorithm to extract keyphrases. To improve the likelihood that the terms extracted are indeed related to the medical domain, we introduced a medical term filtering process to remove any extracted non-medical terms.

### PrefixSpan

The PrefixSpan algorithm was proposed by Pei et al. [14]. PrefixSpan utilises prefix pattern growing, projected database reduction and divide and conquer techniques to perform sequential pattern mining.

#### Problem definition

Let $\mathcal {I} = \{i_1, i_2, ..., i_k\}$ be the set of *k* distinct items, which is often referred to as the alphabet set. An *itemset* is a subset of $\mathcal {I}$ and denoted by ($x_1, x_2, ..., x_n$) where $x_m$ is an item of $\mathcal {I}$. If the itemset only has a single item, the brackets are omitted. A *sequence* is an ordered list of items. A sequence *s* can be denoted by $\langle s_1 s_2 ... s_a\rangle$ where $s_j$ is considered as a single *element*, which is an itemset. $s_i$ is said to occur before $s_j$ for $\forall i\le j$. The length of *s* is the total number of item instances it has. For example, the sequence $\langle ab(adc)\rangle$ has three elements (*a*, *b*, and (*adc*)) and five items, thus it has a length of five. A sequence with *l* instances of items is called a *l*-length sequence. A sequence $\alpha =\langle a_1 a_2 ... a_n \rangle$ is considered a subsequence of sequence $\beta = \langle b_1 b_2 ... b_m\rangle$ and $\beta$ a super sequence of $\alpha$, denoted as $\alpha \sqsubseteq \beta$, if there exists integer $1 \le j_1< j_2< \ldots< j_n\le m$ such that $a_1 \subseteq b_{j_1}, a_2 \subseteq b_{j_2}, ..., a_n \subseteq b_{j_n}$. For example, $\alpha = \langle ab \rangle$ is a subsequence of $\beta = \langle cdabcd\rangle$.

A sequence database *S* can be viewed as a list of $\langle seq\_id, s\rangle$ tuples where $seq\_id$ is the Sequence ID and *s* is a sequence. A tuple $\langle seq\_id, s\rangle$ is said to contain a sequence *t* if *t* is a subsequence of *s*. The support of a sequence *t* is then the number of tuples in the sequence database *S* that contains *t*. A sequence is considered frequent if its support is greater than $min\_sup$, an user defined threshold.

Suppose all items of all elements in a sequence are ordered alphabetically. A sequence $\beta = \langle b_1 b_2 ... b_m\rangle$ is a prefix of the sequence $\alpha = \langle a_1 a_ 2 ...a_n\rangle$ if and only if all the following three conditions hold:

- $$b_i = a_i$$ for $$i\le m - 1$$
- $$b_m \subseteq a_m$$
- All items in ($a_m - b_m$) are alphabetically after those in $b_m$

The postfix of a sequence $$\alpha$$ with respect to a prefix $$\beta$$ is then the sequence in $$\alpha$$ that follows after the prefix $$\beta$$. For example, the sequences $$\langle a\rangle$$, $$\langle ab\rangle$$ and $$\langle aa\rangle$$ are all considered the prefix of the sequence $$\alpha$$$$\langle a(ab)c\rangle$$, but not the sequence $$\langle ac\rangle$$. The postfix of $$\alpha$$ with respect to prefix $$\langle a\rangle$$ is $$\langle (ab)c \rangle$$. The postfix of $$\alpha$$ with respect to prefix $$\langle ab\rangle$$ is $$\langle c\rangle$$. The postfix of $$\alpha$$ with respect to prefix $$\langle aa\rangle$$ is $$\langle (\_b)c\rangle$$. In the last postfix, ($$\_b$$) means that the last element of the prefix $$\langle aa\rangle$$, which is *a*, when joined with *b*, is an element of $$\alpha$$.

For a sequence database *S* and a sequential pattern $\alpha$, the $\alpha$-projected database, denoted as $S|_{\alpha }$, is the list of suffixes of the sequences in *S* with regards to prefix $\alpha$.

In the context of term extraction investigated in this paper, a single word is treated as a single item, and a sentence is a record of a sequence, as such, a document or a collection of documents in our dataset becomes our sequence database. The term extraction task is then converted to find the frequent subsequences (i.e. the single or multiple-word terms) in a sequence database (i.e. the document set).

#### Algorithm

The PrefixSpan algorithm takes a sequence database *S* and the minimum support min_sup as input and outputs the list of frequent sequential patters. The algorithm can be characterised by the recursive function PrefixSpan($\alpha$, *l*, $S|_{\alpha }$), which takes in three parameters: 1) $\alpha$ is the sequential pattern; 2) *l* is the length of $\alpha$; and 3) $S|_{\alpha }$ is $\alpha$-projected database. Initially PrefixSpan($\langle \rangle$, 0, *S*) is called to start the mining process. Given a function call, PrefixSpan($\alpha$, *l*, $S|_{\alpha }$), the algorithm for PrefixSpan works as follows:

1. Scan $S|_{\alpha }$ to find single frequent items, *b*, if either *b* or $\langle b\rangle$ can be appended to $\alpha$ to form a sequential pattern.
2. For each frequent item *b* found, append it to $\alpha$ to create a new sequential pattern $\alpha^{\prime}$ and add $\alpha^{\prime}$ to the final output list of patterns.
3. For each new $\alpha^{\prime}$, construct $\alpha^{\prime}$-projected database $S|_{\alpha^{\prime}}$ and call PrefixSpan($\alpha^{\prime}$, $l+1$, $S|_{\alpha^{\prime}}$)

### C-*Value*

The C-*Value* approach [21] is proposed to recognise domain specific multi-word terms from a corpus. It incorporates both linguistic and statistical information to find these terms.

#### Linguistic component

Linguistic information is used to generate a list of possible candidate terms. This process involves POS tagging and linguistic filtering.

POS tagging is the process of assigning a grammatical tag (e.g. noun, adjective, verb, preposition etc.) to each word in the corpus. After all words in the corpus have been tagged, a linguistic filter can be applied to extract all the candidate terms. The linguistic filter defines the possible sequence of grammatical tags that can formulate a viable term. For example, if we consider a term as a sequence of nouns (i.e. *noun phrase*), a linguistic filter can then be expressed as a rule ($Noun^{+}$) which only permits sequence of nouns to be extracted as possible candidate terms. The choice of filters can affect the overall precision and recall of the candidate list. A ‘closed’ filter typically only permits noun phrases to be extracted as terms. This translates to a higher precision but lower recall. An ‘open’ filter, on the other hand, permits more types of strings to be accepted (e.g. *adjectives* and *prepositions*) as possible candidate terms. This then results in a lower precision but higher recall. For this paper, we choose to use a ‘semi-closed’ filter, which allows *adjectives* and *nouns* to be extracted as terms.

#### Statistical component

Once candidate terms are extracted, they are each evaluated by statistical methods, assigned a termhood (a.k.a. C-*Value*) measure and ranked accordingly, where the highest ranked term being most likely to be a valid term. There are four characteristics of a candidate term that affects its C-*Value*. These are:

- The frequency of the candidate term in the corpus.
- The frequency of the candidate term as part of other longer candidate terms.
- The number of such longer candidate terms.
- The length of the candidate term (as in the number of words).

The C-*Value* of a candidate term *a*, denoted *CV*(*a*) below, depending on whether *a* is a unigram or not, is calculated as follows:

$$CV(a) = {\left\{ \begin{array}{ll} log_{2}|a|\cdot f(a) &{}\text{ unigram } \\ log_{2}|a|\cdot (f(a) - \frac{1}{P(T_{a})} \sum \limits _{b\in T_{a}} f(b)) &{}\text{ otherwise } \end{array}\right. }$$

where *f*(*x*) is the frequency of the term *x* in the corpus, |*a*| is the length of term *a* in number of words, $T_{a}$ is the list of extracted candidate terms containing *a* as a nested term and $P(T_{a})$ is the number of such extracted candidate terms.

### TextRank

**Fig. 1:**
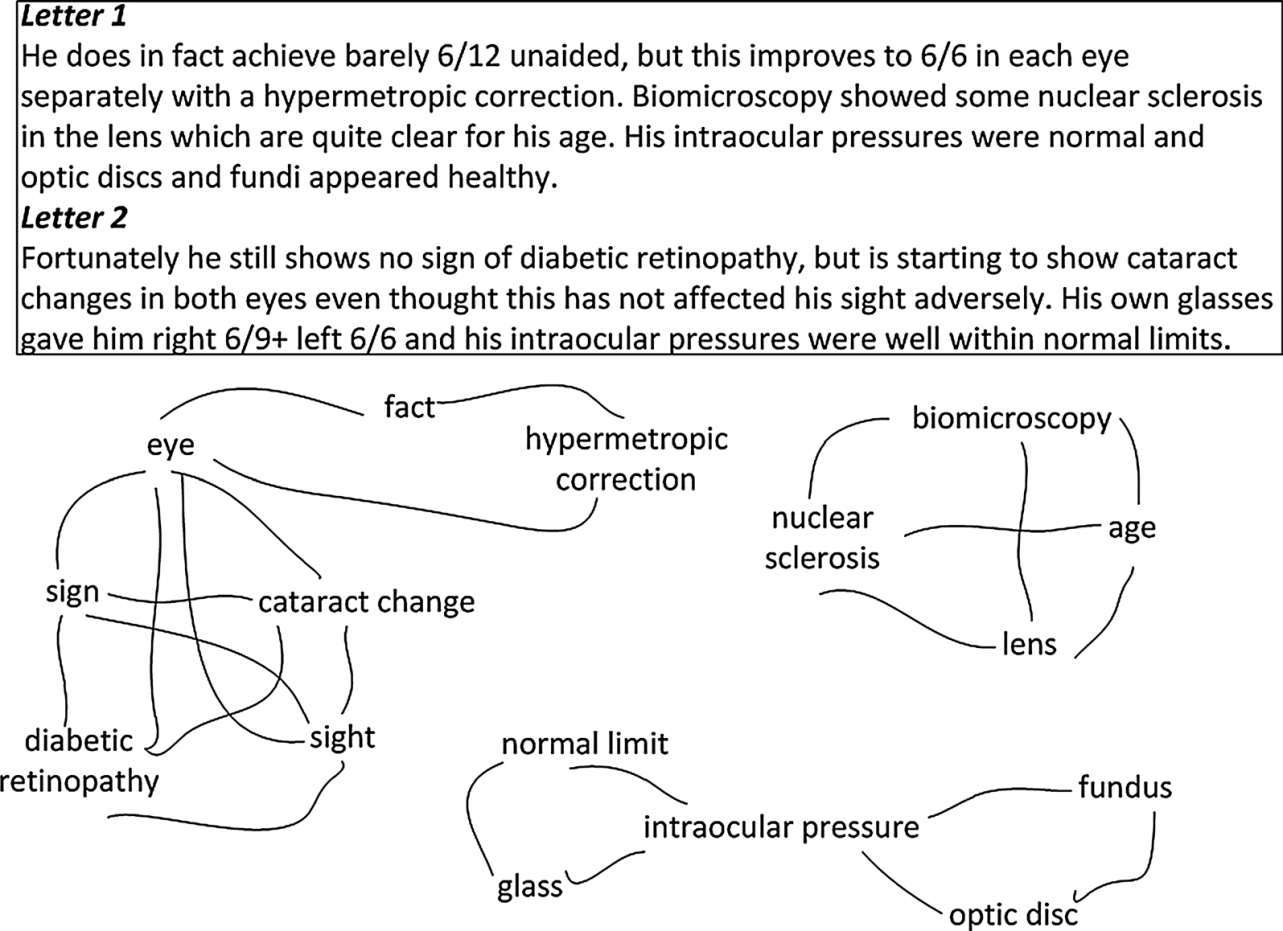
TextRank example graph. The *graph* is created by concatenating two clinical documents. Two terms are connected if they appear in the same sentence

TextRank uses an unweighted undirected graph representing a given text, where words are vertices, and edges denote co-occurrence relations between two words. Two vertices are connected if co-occurrence relations are found within a defined window-size. Figure 1 illustrates the TextRank graph created by concatenating two clinical documents in our dataset.

TextRank implements the concept of ‘voting’. If a vertex $v_i$ links to another vertex $v_j$, then $v_i$ votes for $v_j$; and the importance of $v_j$ increases with the number of votes received. The importance of the vote itself is weighted by the voter’s importance: the more important the voter $v_i$, the more important the vote. The score of a vertex is therefore calculated based on the votes it received and the importance of the voters. The votes received by a vertex can be calculated directly, i.e., the so-called *local vertex-specific information*. The importance of a voter is recursively computed based on both *local vertex-specific information* and *global information*.

TextRank adapts the original PageRank [29] algorithms to calculate word ranks. The original PageRank algorithm works on directed unweighted graphs, $G = (V, E)$. Let $in(v_i)$ be the set of vertices that point to a vertex $v_i$, and $out(v_i)$ be the set of vertices to which $v_i$ point, the score of $v_i$ is calculated by PageRank as:

$$\begin{aligned} S(v_i) = (1-d) + d \times \sum _{j \in in(v_i)} \frac{1}{|out(v_j)|} S(v_j) \end{aligned}$$

In TextRank, the in-degree of a vertex equals to its out-degree, since the graph is undirected. Formally, let *D* denote a document, and *w* denote a word, then $D = \{ w_1, w_2, ... , w_n\}$. The weight of a vertex calculated by TextRanks is:

$$\begin{aligned} WS(v_i) = (1-d) + d \times \sum _{v_j \in in(v_i)} \frac{w_{ji}}{\sum _{v_k \in out(v_j)} w_{jk}} WS(v_j) \end{aligned}$$

where $w_{ij}$ is the strength of the connection between two vertices $v_i$ and $v_j$, and *d* is the dumping factor, usually set to 0.85 [15, 29].

### Medical term filtering

Medical term filtering is the process of removing non-medical terms from the results of C-*Value*, PrefixSpan or TextRank. This is required since non-medical terms can also be extracted by the three algorithms. For example, the term twelve months appear as a valid term in both the output of C-*Value* and PrefixSpan due to its high frequency in the corpus. However, it is not a medical term and thus should not be considered as a valid term.

It is important to note that this process does not guarantee that the terms survived the filtering are actual valid medical terms, but instead it tries to increase the likelihood of the remaining terms being actually medically related.

Due to the sheer volume of terms extracted, it was not feasible for the ophthalmology specialists to do manual filtering. Therefore, in order to facilitate this filtering, we constructed a medical dictionary that contained both practitioner-oriented and consumer-oriented medical terms. Since all of the clinical letters are in the field of ophthalmology, the dictionary contained only ophthalmology-related terms. The dictionary is composed by crawling terms from the websites listed below: 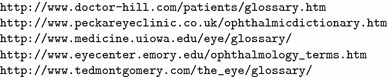


## Genetic algorithm enabled ensemble

Each of the three individual approaches produce a ranking score for a n-gram. After these scores are ordered to indicate the likelihood of being medical terms, we apply a *rank threshold* for each approach as a cut-off to determine whether or not a term is medical related.

In order to combine and maximise the results from the three different techniques, we developed an ensemble medical term extractor by combining the ranking powers of all three methods. Despite being an ensemble of unsupervised methods, we hope it would follow the general observations of ensemble supervised techniques, i.e. ensemble classifiers, which generally outperform individual classifiers [30]. Ensemble classifiers are meta-classifiers that consider the results of a set of primary classifiers using a weighting method or algorithm. In order for an ensemble classifier to outperform its constituent classifiers, the individual classifiers used must be both accurate and diverse [31]. As shown in the “Methodology”, each of the three unsupervised algorithms focuses on different aspects of the language feature space. Sequence mining relies on the fact that complex terms often occur in order, C-*Value* is based on noun phrases, their frequency and the sub-term frequency, whereas TextRank pays more attention to co-occurrence relations from a graph based structure perspective. Therefore, the ensemble unsupervised term extractor should demonstrate better performance than the individual term extractor.

To do this, we combine the individual methods’ ranking scores through a weighted sum into a weighted ranking score $r_w(t)$ for each term *t*, as shown below.

1 $$\begin{aligned} r_w (t) = \sum \limits _{i=1}^n w_i\times r_i(t) \end{aligned}$$

where $w_{1}$ corresponds to the weight assigned to PrefixSpan, $w_{2}$ for C-*Value* and $w_{3}$ for TextRank. Each weight is in the range of [0, 1] and sum up to 1. $r_i(t)$ is the normalised ranking score of term *t* from each algorithm, respectively. The weights are then learnt through a genetic algorithm described below.

### Population

Our population consists of Weights, where each Weight organism contains 3 individual weights corresponding to $w_{1}$, $w_{2}$ and $w_{3}$ as defined above. The capitalised word “Weight” is used hereafter for better clarity to indicate that a Weight is a tuple of three weights.

### Fitness function

To design a sensible fitness function, we need a sensible measurement of performance. First we define the universe ($U=P\cup N$), consisting of both positives (*P*) and negatives (*N*), as a union of filtered terms extracted from all three methods:

$$\begin{aligned} U = \text{ PrefixSpan }\cup \text{ C-}Value\cup\,\, \text{ TextRank } \end{aligned}$$

The positives (*P*) consist of the valid complex terms annotated by the specialists and the unigrams confirmed by the dictionary filtering process.

With the above definition of *U*, *P* and *N* as ground truth, for each method, we can use the standard metrics below to determine precision, recall and F-measure, where

$$\begin{aligned} Precision&= \frac{TP}{TP+FP}\\ Recall&= \frac{TP}{TP+FN}\\ F-measure&= 2\times \frac{Precision\times Recall}{Precision+Recall} \end{aligned}$$

Our fitness function selects the best F-measure. The process for calculating the accuracy of term *t* based on its rank *r* is shown in Algorithm 1.
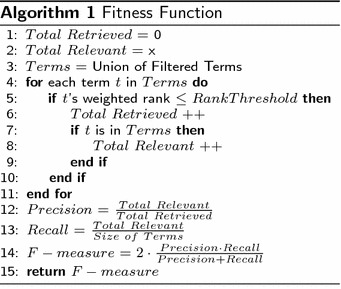


### Crossover

Two different crossover methods are used as explained in more detail below.

#### Naïve crossover

This crossover method is used during early to mid running stages of the genetic algorithm, such as to increase the variability and diversity within the population. The method works as follows: if there are two parent Weights, $P_{1}$ and $P_{2}$, then their children would be $C_{1}$ and $C_{2}$. Then $C_{1}$’s $w_{1}$ ($w_{1}^{C_{1}}$) is equal to the product of $w_{1}$s of both $P_{1}$ and $P_{2}$ normalised to a value within 0 and 1, its $w_{2}$ to the product of $w_{2}$s of both $P_{1}$ and $P_{2}$ normalised to a value within 0 and 1 and its $w_{3}$ to the product of $w_{3}$’s of both $P_{1}$ and $P_{2}$ normalised to a value within 0 and 1. For $C_{2}$, its weights are calculated in the same manner as above, but instead of multiplication, addition of the parents’ individual weights are used, i.e. $C_{2}$’s $w_{1}$ is the sum of $P_{1}$’s $w_{1}$ and $P_{2}$’s $w_{1}$, normalised to a value between 0 and 1, i.e. $w_{1}^{C_{2}}=w_{1}^{P_{1}}+w_{1}^{P_{2}}$.

#### Domination crossover

This crossover method is used during the late running stages of the Genetic Algorithm, in an attempt to maximise the overall fitness of the population. The method works as follows: if there are two parent Weights, $P_{1}$ and $P_{2}$, then their children would be $C_{1}$ and $C_{2}$. To determine $C_{1}$’s weights, the largest *w* from both the parents’ weights is chosen (thus the domination attribute). For example, if $P_{1}$’s $w_{3}$ is the largest among the six possible parents’ weights, then $C_{1}$’s $w_{3}$ is assigned to that value. Then $C_{1}$’s $w_{1}$ and $w_{2}$ is equal to $\frac{1-w_{3}^{P_{1}}}{2}$, to ensure that $C_{1}$’s weights sum up to 1. For $C_{2}$, the same process is repeated as above, the only exception being that the second largest weight is chosen instead.

### Selection

An elitism rate of 40 % for our GA is used. During each generation, the top 40 % *Fittest Weights* of the population are selected to reproduce and create the new population. We have chosen a tournament style approach to reproduction. Four random parents are chosen from the top 40 % Weights to compete in two 2 vs 2 competitions (where the winner is the one with the higher fitness). The winners are then allowed to reproduce and create two children as per the crossover methods above. These reproduction processes continue until new population’s size is 60 % of the original population size. Once the reproduction stage is over, the top 40 % *Fittest Weights* are then added to the new population to create the final new population for the next generation.

### Mutation

We also include mutation to add variability and randomness to the population. We have employed two types of mutation, namely:

- *Gentle mutation* This is where a single random weight *w* of a Weight organism is randomly reassigned a value between 0 and 1 and all the others weights are re-normalised.
- *Super mutation* This is where all the weights of a Weight organism are randomly reassigned a value between 0 and 1 and re-normalised.

During the reproduction stage of each selection process, the children have a 20 % chance of undergoing a gentle mutation. Also, during the reproduction stage, there is also 10 % chance of a Weight organism (within the top *40 % Fittest Weights* of that generation) undergoing a gentle mutation. Super mutation is only used when a new child has one of its weights greater than the domination threshold of 0.85. This is to reduce the possibility of a single Weight organism severely affecting and dominating future generations and populations, due to the domination crossover method we have chosen to use.

## Experiments

### Dataset

We analysed 29,232 clinical letters, written in Microsoft Word, to test the three unsupervised approaches, both separately and together as a GA-enabled ensemble. These letters were written by five different ophthalmology specialists in the past ten years to patients’ General Practitioners. All patients’ names and addresses are removed using anonymisation algorithms we developed for privacy protection before running any of the following experiments.

### Experiment details

#### PrefixSpan

The PrefixSpan implementation provided in the SPMF package [32] was adapted for our frequent single and multi-word terms extraction. The original implementation of PrefixSpan only works for integers and thus we redeveloped the source code for manipulating strings. The modified PrefixSpan requires a sequence file *S* and min_sup as an input, where *S* is a file that contains a single sequence per line (a sequence being equivalent to a single sentence) and min_sup is the minimum frequency for a term to be considered frequent.

We set the minimum support to be 0.1% of the corpus’ size of approximately 30 documents. The minimum pattern length was set to one word (i.e. both single and multi-word terms are considered). The PrefixSpan algorithm was run on the entire corpus.

#### C-*Value*

We developed a preprocessing technique for the C-*Value* program to help identify the candidate terms. It consisted of two steps: text normalising and phrase chunking. In text normalising, we first converted a text into lower-case, and then tokenised and lemmatised the text using Python NLTK [33] tokeniser and lemmatiser. We did not perform stemming because two words with the same stem may have different meanings in medical terminologies.

Phrase chunking was performed by first assigning POS tags for each word using Stanford POS Tagger [34], and then applying heuristics to chunk noun phrases. We considered that a medical term has to be a noun phrase (either a single word noun or multi-words noun phrase) and applied the following heuristics to identify a candidate term: (1) a token with any symbol or punctuation (except hyphen) was treated as invalid; (2) a term should not have more than four tokens; and (3) a term had to match the regular expression $\mathtt {{<}JJ{>}{*}{<}NN.{*}{>}+}$ that looked for a sequence of words that starts with any number of adjectives and ends with one or more nouns.

#### TextRank setup

The strength of TextRank is that it determines the importance of a vertex in terms of both *local vertex-specific information* and *global information*. The *local vertex-specific information* represents how frequently word $w_i$ co-occur with word $w_j$, and the *global information* corresponds to how important the word $w_j$ itself is to the entire text. Thus the word $w_i$ is said to be important when it either has high co-occurrence frequency with $w_j$, or $w_j$ is very important to the text, or both.

However, our corpus consists of relatively short documents, typically around 120 words. After cleaning, each document only contains about 20 candidate terms. Therefore, performing the TextRank over such a short text can only produce trivial result because neither the *local vertex-specific information* nor the *global information* can be well captured. To overcome this issue, we concatenated each of the documents from the entire corpus to build one large document, and then ran the TextRank over this large document. We did this because (1) the actual meaning (the diagnostic information for each patient) contained in each document was not important to the task we were interested in; (2) concatenating documents would not affect the results we wanted to obtain; and (3) concatenating the documents significantly increased the statistical validity of the information used in the ranking algorithm.

In our experiment, two vertices are connected if they co-occur in the same sentence. Initially, the importance of each vertex was uniformly distributed, thus each vertex was assigned an initial value of 1/*n* where *n* is the total number of the vertices in the graph. We also set the damping factor $d=0.85$, iteration to be 30, and threshold of breaking to be 0.0001. To maintain consistency, TextRank uses the same pre-processing process as that of C-*Value*.

The output of PrefixSpan, C-*Value* and TextRank all go through the Medical Term Filtering process in order to increase the likelihood of the final list of terms actually belonging to the medical domain.

#### Genetic algorithm

The following parameters were used in our genetic algorithm, a elitism factor of 40 %, a Children Mutation chance of 20 %, with random mutation of 10 %. The rank threshold was 0.50 while the domination threshold 0.85.

We conducted 100 runs of GA, where each GA run contains 100 organisms and 200 generations.

## Results and discussions

For the evaluation, we have three ophthalmologists annotated the extracted complex terms, including 839 complex terms from PrefixSpan, 2443 from C-*Value* and 2126 from TextRank. Of these complex terms, 181 are common among all three methods, where 27 (15 %) of these common terms are considered as non-domain terms by the doctors based on majority votes. Terms received two votes above are considered valid domain terms.

### Individual algorithms results

The number of terms extracted by PrefixSpan, C-*Value* and TextRank, before and after dictionary filtering are summarised in Table 2. As we can see, the percentage of terms surviving the filtering process from C-*Value* and TextRank are much more than PrefixSpan because they both used noun phrase chuncking as a preprocessing step.

**Table 2:** The number of terms extracted before and after filtering

	PrefixSpan	C-*Value*	TextRank
Before	383,397	56,264	55,055
After	564	3138	3025
Percentage	0.14 %	5.58 %	5.49 %

Tables 3, 4, 5, 6 list the top and bottom 10 terms before and after dictionary filtering. As we can see, the PrefixSpan algorithm generated a lot of short hands notations, and the longest sequence before filtering was “direction of gaze when he was able to open the right eye following the blow out he denies noticing any diplopia”, which is a writing pattern used often by a particular specialist. These frequent sequences retrieved by PrefixSpan, makes it very applicable for writing style analysis. In addition, we observed that PrefixSpan retained more unigrams as compared to the other two methods after filtering (Tables 5, 6). It is also worth noting that the bottom ranked terms from C-*Value* and TextRank still contained sensible terms, indicating low frequency or low co-occurrent terms can still be quite valid domain terms.

**Table 3:** Before filtering top 10

PrefixSpan	C-*Value*	TextRank
Possibly	Right eye	Eye
Possibly a	Left eye	Right eye
F	Month time	Left eye
F She	Intraocular pressure	Left
F FR	Visual acuity	Right
F FR FL	Cataract surgery	Vision
F FR FL N	Optic disc	Review
F FR FL N FR	Current glass	Time
F FR FL N FR FL	Eye	Diagnosis
F Left	Ocular examination	Month

**Table 4:** Before filtering bottom 10

PrefixSpan	C-*Value*	TextRank
sharp in the right	Recent left cataract	Migraine prophylaxis medication
Sharp in the left	Eye institute	Monthly fundus check
sharp in the left eye	Disease for	Upper thorax
Sharp in each	Bar fusion range	Persistent low grade inflammation
Sharp in each eye	Single vision distance	Think mrtaylor
Sharp in each eye with	Sclerotic lens change	Occasional mobic medication
Sharp in each eye with a	Nuclear sclerotic lens change	22 mhg
Sharp pain	Pressure today	Another ct head
Tumour	Titmus stereo	Continued annual pressure check
Stressed	Early nuclear sclerotic lens	Good condtion

**Table 5:** After filtering top 10

PrefixSpan	C-*Value*	TextRank
Eye	Intraocular pressure	Eye
Examination	Visual acuity	Vision
Glasses	Cataract surgery	Diagnosis
Cataract	Optic disc	Examination
Surgery	Eye	Visual acuity
Intraocular	Ocular examination	Intraocular pressure
Acuity	Diabetic retinopathy	Symptom
Cataract surgery	Fundus examination	History
Glaucoma	Vision	Treatment
Lens	Visual field examination	Cornea

**Table 6:** After filtering bottom 10

PrefixSpan	C-*Value*	TextRank
Retinal photocoagulation	Bilateral yag	Pigmentary sign
Homonymous	Intraocular len	Ocular fundus
Detached retina	Simple convergence	Odd microaneurysm
Band	Bilateral normal pressure	Non ischemic retinal vein occlusion
Sphere	Eye surgery	Lens measurement
Refract	External eye	Topical medical treatment
Proliferative	Maddox rod	Blood nose symptom
On examination on visual	External eye disease	Primary diagnosis
Keratic precipitates	Ocular history	Posterior retina
Atypical	Dilated fundus	Posterior chamber lens implant

### GA Results

The extraction results in Tables 3, 4, 5, 6 confirmed our hypothesis that the three different techniques are complementary. As shown in Tables 7 and 8, individually, TextRank’s performance is similar to C-*Value*. They both outperform PrefixSpan on all accounts.

**Table 7:** Performance measures with unigram

	Prefix (%)	C-*Value* (%)	TextRank	GA (avg)
Precision	59.30	61.25	*63.83*	
Recall	5.36	*82.30*	75.36	
F-Measure	9.82	*70.24*	69.12	*72.47* %

**Table 8:** Performance measures without unigram

	Prefix (%)	C-*Value* (%)	TextRank (%)	GA (avg)
Precision	18.09	49.93	*51.60*	
Recall	5.36	*82.30*	81.98	
F-Measure	8.27	62.16	*63.34*	*65.65* %

Table 9 shows the weights of two separate runs of GAs with and without unigrams. As we can see, PrefixSpan dominated the final ranking score, especially when single word medical terms (unigrams) are taken into consideration, with a very high weight of 92.84 %. In the case of complex multi-word medical phrases, TextRank acquire higher weights than C-*Value*. This seems to be counter-intuitive when traditional ensemble approaches tend to lean towards better performed methods. In this case, please note that the positive terms are a union of all three methods and the overlap of common terms are a small proportion of the total number of terms extracted. Therefore, the GA generated weights is pushing the PrefixSpan terms into higher ranks when they are available, because they constitute a small portion of the universe. In the case of complex terms, the PrefixSpan results only constitutes 15.51 % of the total number of complex terms generated.

**Table 9:** Weights of GA-enabled ensemble (averaged over 100 runs)

$w_i$	PrefixSpan (%)	C-*Value* (%)	TextRank (%)
With unigrams	92.84	0.28	6.88
Without unigrams	82.96	0.1	16.94

**Fig. 2:**
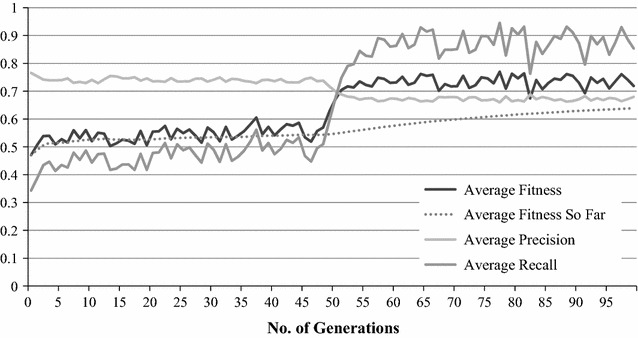
GA optimisation process with unigram

**Fig. 3:**
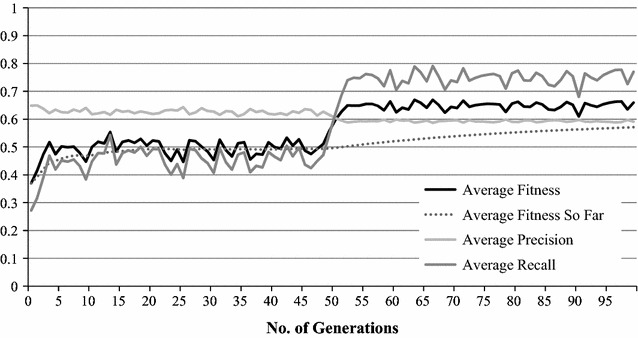
GA optimisation process without unigram

The fitness values (in this case, F-measure) were calculated by averaging the best, worst and average fitness achieved by the population at the end of each run over the entire 100 runs. Likewise, the Fittest Weight’s values are the average of the weights of all the Fittest Weight at the end of each run over the entire 100 runs. Results are shown in Figs. 2 and 3.

The top and bottom 20 terms extracted using the GA weighted score, with and without unigrams are shown in Tables 10 and 11, respectively.

**Table 10:** GA top 20

With unigrams	Without unigrams
Eye	Cataract surgery
Examination	Intraocular pressure
Cataract	Visual acuity
Surgery	Macular degeneration
Cataract surgery	Contact lens
Glaucoma	Optic disc
History	Intraocular lens
Lens	Posterior vitreous detachment
Diagnosis	Anterior chamber
Acuity	Cataract extraction
Pressure	Dry eye
Macula	Retinal detachment
Cornea	Visual field
Pterygium	Double vision
Diplopia	Optic nerve
Contact	Posterior capsule
Tear	Retinal vein occlusion
Macular degeneration	Colour vision
Intraocular pressure	Fluorescein angiography
Angle	Meibomian gland dysfunction

**Table 11:** GA bottom 20

With unigrams	Without unigrams
Bilateral defect	Conjunctival naevus
de	Chronic simple glaucoma
cl	Central visual field test
os	Blepharo spasm
Visual test	Bilateral posterior uveitis
Vision bilateral	Bilateral macular pattern dystrophy
Pupillary conjunctivitis	Bilateral iritis
Normal migraine	Atypical migraine
Macula i	Atropine occlusion
Jaw wink	Acute iritis
Inferior retinal break	Active epithelial disease
i o p	Macular microaneurysms
Diplopia n	Haptic lens
i	Choroidal naevi
uv	Senile ptosis
al	Lacrimal pressure
od	Arteritic ischaemic optic neuropathy
Arteritic ischaemic optic neuropathy	Choroidal neovascular
Inferior hemi retinal vein occlusion	Inferior hemi retinal vein occlusion
Eye i	Eye i

## Conclusion

In this research, we conducted intensive medical term extraction exercise using a real-world document set of near 30,000 clinical letters collected over the past 10 years from one eye clinic. We used three popular ranking algorithms for unsupervised medical term extraction, namely, PrefixSpan, C-*Value* and TextRank, as each covered different aspects of the language feature space. A genetic algorithm was developed to generalise the weight learning process by linearly combining the three ranking scores in an ensemble. The experiments showed promising results that with minimal amount of annotated data, an GA-enabled ensemble of unsupervised approaches can achieve an average F-measure of 65.65 % when considering only complex medical terms, and a F-measure of 72.47 % if we take single word terms (i.e. unigrams) into consideration.

As side products of this research, we also developed algorithms and strategies for anonymising medical letters and constructing online dictionaries using web resources, which are not detailed in this paper due to lack of space and lack of immediate relevance.

The promising results confirm our system can be used as a solid foundation for bootstrapping of supervised medical entity extraction. On the other hand, it also poses a number of interesting research questions that are worth pursuing. More immediately, we will investigate the validity of bottom ranked terms and incorporate doctors’ annotations through semi-supervised learning to further improve performance.

## Footnotes

1 http://metamap.nlm.nih.gov.

2 https://uts.nlm.nih.gov/home.html.

3 Mosman Park, Western Australia, Australia.

4 http://www.nlm.nih.gov/research/umls/Snomed/snomed_main.html.
